# A Systematic Review of the Quality of IV Fluid Therapy in Veterinary Medicine

**DOI:** 10.3389/fvets.2017.00127

**Published:** 2017-08-14

**Authors:** William W. Muir, Yukie Ueyama, Jessica Noel-Morgan, Allison Kilborne, Jessica Page

**Affiliations:** ^1^QTest Labs, Columbus, OH, United States; ^2^College of Veterinary Medicine, Lincoln Memorial University, Harrogate, TN, United States; ^3^Center for Cardiovascular and Pulmonary Research, The Research Institute at Nationwide Children’s Hospital, Columbus, OH, United States; ^4^Department of Veterinary Clinical Sciences, College of Veterinary Medicine, Columbus, OH, United States

**Keywords:** intravenous fluids, crystalloids, colloids, companion animals, cattle

## Abstract

**Objective:**

To evaluate the quality of the veterinary literature investigating IV fluid therapy in dogs, cats, horses, and cattle.

**Design:**

Systematic review.

**Procedures:**

The preferred reporting of items for systematic review and meta-analysis protocols (PRISMA-P) was employed for systematic review of all relevant IV fluid therapy manuscripts published from January 1969 through December 2016 in the Commonwealth Agricultural Bureaux International (CABI) database. Independent grading systems used to evaluate manuscripts included the updated CONsolidated Standards of Reporting Trials 2012 checklist, risk of bias for animal intervention studies, criteria for levels of evidence, and methodological quality (Jadad scale). The quality of articles published before and after 2010 was compared.

**Results:**

One hundred and thirty-nine articles (63 dogs, 7 cats, 39 horses, 30 cattle) from 7,258 met the inclusion criteria. More than 50% of the manuscripts did not comply with minimal requirements for reporting randomized controlled trials. The most non-compliant items included identification of specific predefined objectives or a hypothesis, identification of trial design, how sample size was determined, randomization, and blinding procedures. Most studies were underpowered and at risk for selection, performance, and detection bias. The overall quality of the articles improved for articles published after 2010.

**Conclusion and clinical relevance:**

Most of the veterinary literature investigating the administration of IV fluid therapy in dogs, cats, horses, and cattle is descriptive, does not comply with standards for evidence, or provide adequate translation to clinical practice. Authors should employ and journal editors should enforce international consensus recommendations and guidelines for publication of data from animal experiments investigating IV fluid therapy.

## Introduction

Intravenous fluid therapy is prescribed as therapeutic treatment for most critically ill animals. Crystalloids, especially lactated Ringers solution and 0.9% saline (NS), and to a lesser extent colloids are administered with the primary goal of maintaining or restoring vascular volume and tissue perfusion. Intravenous fluids, crystalloids or colloids, produce varying effects on the extracellular fluid dependent upon their composition, tonicity, caloric, acid–base, hemostatic, rheological, and immunologic effects (1–5). Dissimilarities in IV fluid composition and the volume of IV fluid administered have resulted in serious debates, condemnations, and warnings of select IV fluid solutions (6–12). Standardized or “one size fits all” protocols for IV fluid therapy have been abandoned for the treatment of patient-specific (context sensitive: hypotension, hemorrhage, trauma, sepsis) fluid administration procedures guided by goal-directed therapy (13–16). Large preclinical and clinical human trials buoyed by data generated from various experimental animal (rodent, canine, swine) models are generally considered to provide the best evidence for current recommendations (15, 17, 18). Systematic reviews of animal trials investigating IV fluid therapy in controlled and uncontrolled hemorrhage published in PubMed, Medline, Embase, Scopus, and The Cochrane Library have concluded that, although animal experiments are essential for human health, their results are underpowered and suffer from substantial heterogeneity, model dependency, and bias (17–20). We conducted a systematic review of animal trials that investigated IV fluid therapy and were published in the Commonwealth Agricultural Bureaux International (CABI) database in order to determine their compliance with current standards of evidence (21). The CABI database was chosen because it provided a larger number of veterinary citations than the previously identified databases. We evaluated whether or not citations investigating IV fluid therapy complied with documented grading systems for methodological quality, minimal requirements for randomized controlled trials (RCTs), methodologies employed for the elimination of bias, and clinical relevance (21–24).

## Materials and Methods

A search of the CABI (8,493 total serials; 7,532 global health serials; 212 veterinary specific serials) database from January 1969 through December of 2016 was performed to identify suitable articles. Titles that included the IV administration of crystalloids, colloids, and albumin in dogs, cats, horses, and cattle were evaluated for potential review. The primary search terms included intravenous fluid therapy, fluid resuscitation, fluid bolus, fluid challenge, crystalloid, saline, 0.9% sodium chloride, hypertonic saline, Ringers, lactated Ringer, Hartman’s solution, acetated Ringer solution, polyionic solution, Plasmalyte, Normosol, colloid, plasma substitute, hyroxyethyl starch, hetastarch (Hespan^®^, Hextend^®^), tetrastarch (Voluven^®^, Vetstrarch^®^), pentastarch, dextran, and albumin. All possible combinations and permutations of the search terms were examined. Blood, blood products, and gelatins, other than albumin, were not included. Manuscripts published in English, German, Japanese, Portuguese, French and Spanish were included. Retrospective studies, duplications, review articles, abstracts, single animal case reports, and *in vitro* experiments were excluded from analysis. Studies investigating alternate routes of fluid administration other than IV and species other than those identified above were also excluded. The bibliographies of 186 selected manuscripts were assessed in order to identify additional manuscripts that met inclusion criteria. The review process employed criteria for preferred reporting of items for systematic review and meta-analysis protocols (PRISMA-P) (25). Review and data extraction were performed by the study participants using four independent grading systems that included the updated CONsolidated Standards of Reporting Trials (CONSORT) 2012 checklist (25 items including identification of a control group), risk of bias (RoB) for animal intervention studies (SYRCLE’s RoB tool), criteria for levels of evidence [Center for Evidence-Based Medicine (CEBM): http://cebm.com; 2011], and the Jadad scale (21, 22, 24). The raters were trained to review and grade manuscripts and medical literature. A Fleiss’ Kappa statistic was performed after training and indicated almost perfect agreement (0.8) among raters. The disposition of all articles was subsequently analyzed in order to assess their compliance with key requirements for reporting RCTs (23). These requirements overlapped with and incorporated 15 specific CONSORT statement items that included specific objectives or a hypotheses (item 2b), trial design including a control group (item 3c), allocation ratio (item 3a), important changes to the methods after trial commencement (item 3b), participant eligibility criteria (item 4a), pre-definition of primary and secondary outcomes (item 6a), how sample size was determined (item 7a), random allocation procedures (item 8a), blinding (item 11a), statistical methods (items 12a, 12b), number of participants as assigned (item 16), precision of primary and secondary outcome results (item 17a), all harms (item 19), generalizability of findings (item 21), and interpretation (item 22). The compliance of manuscripts published before and after 2010 using the 15 specific CONSORT items for reporting RCTs was also determined as were articles that specifically stated they were randomized and controlled (23). We intentionally selected year 2010 as a cutoff date based upon publications published in 2010 that specifically addressed multiple “guidelines” for reporting research trials (25–28). Data are presented as the nearest percentage in order to estimate overall compliance.

## Results

A total of 7,258 manuscripts were identified that contained one or more primary search terms. Of these, 4,284 manuscripts were eligible for further review. One hundred and eighty-six manuscripts met all inclusion requirements. An additional 47 manuscripts were excluded because they did not comply with all entry criteria (Table 1). A total of 139 articles (63 dogs; 7 cats; 39 horses; 30 cattle) were analyzed for compliance with CONSORT statement recommendations (Table 1). Fifteen trials in dogs, 2 trials in cats, 5 trials in horses, and 3 trials in cattle were performed during sedation and/or anesthesia. The four most frequent reasons for investigating IV fluid therapy were as follows: sepsis/endotoxemia (22 articles); hemodynamic, biochemical, or acid–base effects (19 articles); diarrhea (17 articles); and hemorrhage and/or hypotension (15 articles). Only nine (3 dogs; 1 cat; 2 horses; 3 cattle) articles included predefined criteria for determining morbidity and mortality. Items that were the most non-compliant (<50%) with CONSORT recommendation statements included: identified as a randomized trial in the title (item 1a); specific objectives or a hypothesis (item 2b); important changes to the methods after trial commencement (item 3b); pre-specified primary and secondary outcomes (item 6a); how sample size was determined (item 7a); method used to generate the random allocation (item 8a); type of randomization: details and restrictions (item 8b); who generated the random allocation sequence, who enrolled participants, and who assigned participants to interventions (item 10); blinding and who was blinded after assignment to an intervention and how (item 11a); methods for additional analyses, such as analyses and adjusted analyses (item 12b); table showing baseline demographic and clinical characteristics for each group (item 15); results of additional analyses performed, including subgroup analyses and adjusted analyses, distinguishing pre-specified from exploratory (item 18); where the full trial protocol can be accessed, if available (item 24); and, sources of funding or other support (item 25) (Table 2). Notably, only 4% of the articles were blinded and 17% of the articles were registered (Table 2; item 24). Most of the articles were controlled but less than 15% of all articles were randomized providing an Oxford Center for Evidence-based Medicine Level of Evidence grade of 3 or greater.

**Table 1:** Flow diagram of search strategy.

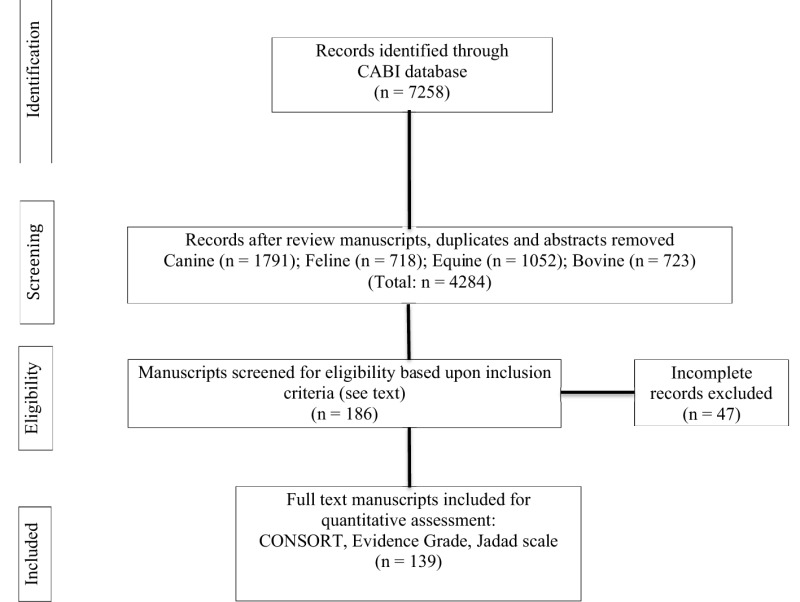

**Table 2:** Compliance of 139 IV fluid therapy articles with CONsolidated Standards of Reporting Trials (CONSORT) guidelines.

CONSORT item	Item number/item statement	Compliance (%)
Title and abstract	la: Identified as a randomized trial in the title	1
	lb: Structured abstract	65
Background and objectives	2a: Scientific background and rationale	99
	2b: Specific objectives or hypotheses	47
Trial design	3a: Description of trial design (such as parallel, factorial) including allocation ratio	45
	3b: Important changes to methods after trial commencement (such as eligibility criteria), with reasons	12
	3c: Control group	73
Participants	4a: Eligibility criteria for participants	90
	4b: Settings and location for data collection	92
Interventions	5: The interventions for each group with sufficient details to allow replication, including how and when they were actually administered	88
Outcomes	6a: Pre-specified primary and secondary outcomes	45
	6b: Changes to trial outcomes after study initiation	30
Sample size	7a: How sample size was determined	4
	7b: Interim analysis when applicable; stopping guides	NA
**Randomization**	
Sequence	8a: Method used to generate the generation random allocation sequence	14
	8b: Type of randomization: details and restrictions	9
Allocation concealment mechanism	9: Mechanism for implementing random allocation	13
Implementation	10: Who generated the random allocation sequence, who enrolled participants, and who assigned participants to interventions	0
Blinding	11a: If done, who was blinded after assignment to interventions (for example, participants, care providers, those assessing outcomes) and how	4
	l1b: Description of similarity of interventions, if relevant	1
Statistical methods	12a: Statistical methods used to compare groups for primary and secondary outcomes	88
	12b: Methods for additional analyses, such as analyses and adjusted analyses	29
Participant flow	13a: For each group, the numbers of participants who were randomly assigned, received intended treatment, and were analyzed for the primary outcome	88
	13b: Subject losses and exclusions, with reasons	57
Recruitment	14a: Dates of recruitment and follow-up	36
	14b: Why the trial ended or was stopped	20
Baseline data	15: Table showing baseline demographic and clinical characteristics for each group	30
Numbers analyzed	16: For each group, number of participants (denominator) included in each analysis and whether the analysis was by original assigned groups	84
Outcomes and estimation	17a: For each primary and secondary outcome, results for each group, and the estimated effect size and its precision (such as 95% confidence interval)	78
	17b: Absolute and relative effect size for binary data	38
Ancillary analyses	18: Results of additional analyses performed, including subgroup analyses and adjusted analyses, distinguishing pre-specified from exploratory	28
Harms	19: All important harms or unintended effects in each group	75
Limitations	20: Trial limitations (addressing sources of potential bias, imprecision, multiplicity of analyses)	55
Generalizability	21: Generalizability (external validity, applicability) of the trial findings	99
Interpretation	22: Interpretation consistent with results, balancing benefits and harms, and considering relevant evidence	96
Registration	23: Registration number and name of trial registry	17
Protocol	24: Where the full trial protocol can be accessed, if available	4
Funding	25: Sources of funding or other support	46
Jadad score		0.99

Compliance of the 139 articles with CONSORT items considered fundamental for conducting a RCT (items 2b, 3a, 3b, 3c, 4a, 5, 6a, 7a, 8a, 11a, 12a, 16, 17a, 19, 21, 22) varied for each item among the different species (Table 3) (22). Most articles (>80%) provided statements describing: eligibility criteria for participants (item 4a); the interventions for each group with sufficient details to allow replication, including who and when administered (item 5); statistical methods used to compare groups for primary and secondary outcomes (item 12a); generalizability of the trial findings (item 21); and interpretations consistent with results (item 22) (Table 3). The overall and individual species Jadad scores were 0.99 (overall) and 1.03 (dogs), 1.29 (cats), 1.08 (horses), and 0.70 (cattle) (Table 3). Compliance (%) of articles with CONSORT items considered fundamental for conducting a RCT, and the Jadad scores generally improved after 2010 (Table 4). Compliance with CONSORT items considered fundamental for conducting a RCT were improved (by >50%) the most for select items: specific objectives or hypotheses (item 2b); description of trial design including allocation ratio (item 3a); pre-specified primary and secondary outcomes (item 6a); how sample size was determined (items 7a); sequence generation and method used to generate the random allocation sequence (item 8a); who was blinded after assignment to interventions and how (item 11a); and methods for additional analyses, such as analyses and adjusted analyses (item 12a) and methods for additional analysis (item 12b). Overall compliance remained low (<50%) for items: important changes to methods after trial commencement (item 3b); how sample size was determined (item7a); sequence generation: method used to generate the random allocation sequence (item 8a); and methods for additional analyses, such as analyses and adjusted analyses (item 12b) (Table 4).

**Table 3:** Compliance (%) of 139 IV fluid therapy articles with key CONsolidated Standards of Reporting Trials (CONSORT) guidelines for reporting RCTs.

CONSORT item	Item number/item statement	D (63)	C (7)	H (39)	Ca (30)	O (139)
Background and objectives	2b: Specific objectives or hypotheses	55	67	51	23	47
Trial design	3a: Description of trial design including allocation ratio	48	43	54	30	45
	3b: Important changes to methods after trial commencement	10	29	18	4	12
	3c: Control group	75	86	79	57	73
Participants	4a: Eligibility criteria for participants	89	100	95	83	90
Interventions	5: The interventions for each group with sufficient details to allow replication, including who and when administered	90	71	92	83	88
Outcomes	6a: Pre-specified primary and secondary outcomes	57	29	54	13	45
Sample size	7a: How sample size was determined	2	14	10	0	4
Randomization	8a: Sequence generation: method used to generate the random allocation sequence	10	29	18	17	14
Blinding	11a: Who was blinded after assignment to interventions and how	<1	<1	7	<1	4
Statistical methods	12a: Statistical methods used to compare groups for primary and secondary outcomes	90	86	90	83	88
	12b: Methods for additional analyses, such as analyses and adjusted analyses	29	14	31	30	29
Numbers analyzed	16: For each group, number of participants included in each analysis and whether the analysis was by original assigned groups	84	71	92	77	84
Outcomes and estimation	17a: For each primary and secondary outcome, results for each group, and the estimated effect size and its precision	86	100	72	63	78
Harms	19: All important harms or unintended effects in each group	73	100	72	77	75
Generalizability	21: Generalizability of the trial findings	97	100	100	100	99
Interpretation	22: Interpretation consistent with results	95	100	95	97	96
Jadad scores		1.03	1.29	1.08	0.70	0.99

*RCT, randomized controlled trial; (), no. manuscripts; D, dog; C, cat; H, horse; Ca, cattle; O, overall*.

**Table 4:** Compliance (%) of 139 IV fluid therapy articles with key CONsolidated Standards of Reporting Trials (CONSORT) guidelines for reporting RCTs, before and after 2010.

CONSORT item	Item number/item statement	<2010 (94)	≥2010 (45)	O (139)
Background and objectives	2b: Specific objectives or hypotheses	39	66	47
Trial design	3a: Description of trial design including allocation ratio	32	73	45
	3b: Important changes to methods after trial commencement	10	18	12
	3c: Control group	73	71	73
Participants	4a: Eligibility criteria for participants	85	100	90
Interventions	5: The interventions for each group with sufficient details to allow replication, including who and when administered	87	91	88
Outcomes	6a: Pre-specified primary and secondary outcomes	34	69	45
Sample size	7a: How sample size was determined	1	11	4
Randomization:	8a: Sequence generation: method used to generate the random allocation sequence	6	31	14
Blinding	11a: Who was blinded after assignment to interventions and how	0	4	4
Statistical methods	12a: Statistical methods used to compare groups for primary and secondary outcomes	86	93	88
	12b: Methods for additional analyses, such as analyses and adjusted analyses	21	44	29
Numbers analyzed	16: For each group, number of participants included in each analysis and whether the analysis was by original assigned groups	82	89	84
Outcomes and estimation	17a: For each primary and secondary outcome, results for each group, and the estimated effect size and its precision	73	86	78
Harms	19: All important harms or unintended effects in each group	73	78	75
Generalizability	21: Generalizability of the trial findings	100	96	99
Interpretation	22: Interpretation consistent with results	96	96	96
Jadad scores		0.73	1.51	0.99

*RCT, randomized controlled trial; (), no. manuscripts; O, overall*.

Fifty-five articles specifically stated they were randomized and controlled. Thirty-one articles were published before (dog 13, cat 3, horse 4, cattle 11) and 24 articles after (dog 12, cat 0, horse 9, cattle 3) 2010. Compliance (%) of these articles with select CONSORT statement items considered fundamental for conducting a RCT, and their Jadad scores were generally greater compared with all 139 articles. Compliance improved even more for articles published after 2010 (Table 5).

**Table 5:** Compliance (%) of 55 IV fluid therapy articles stating they were controlled and randomized with key CONsolidated Standards of Reporting Trials (CONSORT) guidelines for reporting RCTs, before and after 2010.

CONSORT item	Item number/item statement	<2010 (31)	≥2010 (24)	O (55)
Background and objectives	2b: Specific objectives or hypotheses	58	71	64
Trial design	3a: Description of trial design including allocation ratio	55	75	64
	3b: Important changes to methods after trial commencement	16	8	13
	3c: Control group	100	100	100
Participants	4a: Eligibility criteria for participants	90	100	95
Interventions	5: The interventions for each group with sufficient details to allow replication, including who and when administered	94	88	91
Outcomes	6a: Pre-specified primary and secondary outcomes	42	71	55
Sample size	7a: How sample size was determined	3	21	11
Randomization	8a: Sequence generation: method used to generate the random allocation sequence	16	54	33
Blinding	11a: Who was blinded after assignment to interventions and how	0	17	7
Statistical methods	12a: Statistical methods used to compare groups for primary and secondary outcomes	97	96	96
	12b: Methods for additional analyses, such as analyses and adjusted analyses	19	42	29
Numbers analyzed	16: For each group, number of participants included in each analysis and whether the analysis was by original assigned groups	77	92	84
Outcomes and estimation	17a: For each primary and secondary outcome, results for each group, and the estimated effect size and its precision	90	88	89
Harms	19: All important harms or unintended effects in each group	81	83	82
Generalizability	21: Generalizability of the trial findings	100	96	98
Interpretation	22: Interpretation consistent with results	100	92	96
Jadad scores		1.45	2.08	1.73

*RCT, randomized controlled trial; (), no. manuscripts; O, overall*.

## Discussion

This is the first systematic review of the CABI database that has evaluated articles investigating IV fluid administration in dogs, cats, horses and cattle. Two percent (139) of the 7,258 fluid therapy manuscripts published in veterinary journals listed in the CABI database met our criteria for being categorized as IV fluid therapy. Of these, more than 50% of the manuscripts did not comply with CONSORT statement guidelines describing trial design, sample size determination, methods of randomization or blinding. Only 20 articles (14%) described randomization methods. Manuscripts published after 2010 had a greater tendency to comply with CONSORT statement guidelines than those published before 2010 but no manuscript complied with all requirements. Importantly, only 45% of the articles described trial design (i.e., parallel; crossover) and fewer yet (12%) described changes to the methods (e.g. eligibility criteria).

Multiple search methods and evaluative procedures have been developed for determining the quality, validity, and relevance of experimental and clinical research in humans and animals (21–26, 29–31). The CONSORT guideline was developed to improve the reporting of RCTs, so that readers would be able to understand trial design, analysis, and interpretation in order to assess the validity of the results. The CONSORT scoring system includes a 25-item checklist that provides guidance for reporting focusing on common experimental or clinical designs (21). The CEBM “levels of evidence” was introduced in 1998 to enhance the finding of appropriate evidence, and the Jadad scale was developed to assess the quality of published clinical trials based upon randomization, blinding, and an accounting of all subjects selected after admission to the study (24, 31). The low overall Jadad score (0.99; Table 3) for the 139 articles in our study can be attributed to the low percentage of manuscripts that were randomized and blinded.

Defining the primary research question, proper trial design with predefined outcome measures, sample size determination, and appropriately planned statistical approaches are interrelated CONSORT statement items that should be determined prior to study execution (17, 32–34). Of the 139 manuscripts reviewed in the present study, more than 50% failed to state specific objectives or hypotheses, disclose proper description of trial design, or predefine primary outcome measures (Table 2; items 2b, 3a, 6a). Trial design features that directly impact the statistical plan include allocation ratio (not all studies use 1:1) and type (e.g., parallel, multi-arm parallel, factorial, crossover, cluster), in addition to whether the study seeks to determine superiority, equivalence, or non-inferiority of different interventions (item 3) (35, 36). Although improvement in each of these items was observed in articles published after 2010, approximately one-third of the studies still failed to satisfy these criteria (Table 4). It was noted that statistical test selection was often poorly described and did not address test assumptions or the basis for employing parametric or other statistical approaches. Beyond primary and secondary outcomes, our study determined a lower rate of additional analyses (29%; Table 2) consistent with investigations with limited sample sizes that are focused on a restricted number of variables (37).

One of the most troubling results from the present investigation was the negligible number of studies (4%; Table 2) that disclosed sample size calculation. This oversight could be due to a lack of awareness of its true importance, lack of demand by an IACUC, or because it was not required by the journal upon submission. Regardless of cause, the means for determining the number of subjects/observations included carries pivotal statistical importance, directly related to study design, power, elimination of bias, and ultimately, the application of findings to a larger population. Only an adequate sample size will yield enough power effectually reduce the chance of type I and type II errors (i.e., false-positive or false-negative results) (34, 37) and prevent erroneous results (34, 38, 39). Accurate reporting of methods, including the means by which sample size was determined, should be mandatory for the proper assessment of the validity of the results and the conclusions drawn (37, 40). Moreover, although the significance level was often disclosed (typically 0.05), there was rarely mention of a targeted statistical power. Despite this, 88% (12a) of the studies disclosed the statistical methods employed for analyses of the main outcomes reported (93% after 2010; Table 4) and 78% stipulated estimates of effect size (Table 2). Importantly, *post hoc* power calculations risk bias. Furthermore, some results may have reached statistical significance had a larger sample size been employed (37–40). Secondary outcomes, on the other hand, may not necessarily require prospective planning, however, the method and timing for their selection and evaluation should be described. This allows the reader to correctly differentiate potentially promising results from those requiring further hypothesis testing before generalizing conclusions to a larger population (30, 40).

The choice of subject allocation or failure to disclose the kind of randomization did not preclude the inclusion of manuscripts from the present study. Few studies (14% Table 2) specified the method by which this was done and only 9% (13/139) were randomized appropriately. Only five articles (4%) mentioned who was blinded and only two of these described the method employed for blinding. The CONSORT criteria questions whether, *if* blinded, *who* was blinded and *how* (11a, 11b; Table 2). Blinding is essential but not always feasible for clinical trials that compare interventions. Depending on the characteristics of the intervention, blinding may be difficult to implement. The technique and people involved in blinding are important factors for eliminating both intentional and non-intentional bias linked to each individual researcher’s evaluation or expectation, especially when less objective observations are utilized (27, 30, 41). None of the articles analyzed complied with all SYRCLE criteria. All studies were at risk of selection, performance, and detection bias from a lack of reporting of methods for generating (14%) and implementing (9%) randomization and blinding procedures, respectively (Table 2). All studies were also at risk of detection bias due to a lack of reporting of systematic differences between or among groups on how and by whom outcomes were determined. Unlike random error caused by sampling variability and a small sample size, or both, bias is independent of both sample size and statistical significance (27, 30).

Generalizability is determined by many of the same criteria employed to determine internal and external validity and includes sample selection, hypothesis testing, effect size measures, standards of efficacy, elimination of bias and confounding (unaccounted variables), and experimental reproducibility (42, 43). Replication determines whether the study results are likely to apply, generally or specifically, in comparable settings (externally valid). However, less than 50% of the overall manuscripts, regardless of species, provided sufficient detail to enable replication of the experimental procedures (Tables 2 and 3). These findings suggest that less than half of the manuscripts published in the veterinary literature are repeatable or transferable to a larger subset of the animal population.

We were unable to perform a meta-analysis for the studies included in this review due to differences in experimental designs, outcome measures, and statistical approaches. We intentionally selected the CONSORT and Jadad evaluation tools because of their popularity, although alternative guidelines for conducting and reporting *in vivo* animal experiments have been proposed (26, 28, 44–47). Notably, the Jadad scoring system has been criticized for being too simplistic and placing too much weight on blinding, in addition to low consistency between different raters (48). We also had difficulty applying the complete Jadad scale (methodological quality of a clinical trial) since most of the manuscripts were not randomized nor stated a method for randomization, if randomized. The Jadad scoring system grants a point if a study claims to have been randomized but removes a point if the description of randomization leads to the conclusion that it was inappropriately done. In the absence of a description for a randomization technique, we maintained one point if randomization was stated.

## Conclusion

Systematic reviews of experimental and preclinical research trials identified in PubMed have questioned whether animal experimentation informs human healthcare based upon small sample size and statistical heterogeneity among the different experiments (17–20, 47). Our study identified major areas of non-compliance with consensus recommendations for the quality and clinical relevance of IV fluid therapy studies published in journals listed in CABI. Major areas of non-compliance included identification of predefined outcome variables, sample size determination, randomization, and blinding. The majority of the veterinary literature investigating the administration of IV fluid therapy in dogs, cats, horses, and cattle is descriptive and does not comply with current evidence standards nor does it provide adequate translation to veterinary clinical practice or to human health. Clinical and experimental animal research programs should provide educational programs that emphasize compliance with current standards for animal care and use and standards of evidence. Journal publishing organizations should provide webinars, education materials, and check lists that inform and explain the fundamental components of good evidence. Journal editors should implement and enforce international evidence-based medicine consensus guidelines for publication of animal studies if future research is to substantially contribute to animal (and possibly human) health care and welfare (49, 50).

## Author Contributions

WM, YU, JN-M, and AK contributed to the analysis, interpretation, and writing of this review. WM and AK searched the Commonwealth Agricultural Bureaux International database, organized, analyzed, and abstracted all manuscripts. JP organized the search terms and programmed the computer search for all selected terms.

## Conflict of Interest Statement

The authors declare that the research was conducted in the absence of any commercial or financial relationships that could be construed as a potential conflict of interest. The reviewers, NP and JH, and handling Editor declared their shared affiliation, and the handling Editor states that the process nevertheless met the standards of a fair and objective review.
